# Commentary on ‘Prognostic factors for acute vertebrobasilar artery occlusion-reperfusion: a multicenter retrospective cohort study’

**DOI:** 10.1097/JS9.0000000000001668

**Published:** 2024-05-22

**Authors:** Jinping Zhang, Haitao Li, Zunlu Zhang, Mingzhe Hu, Bingchen Li

**Affiliations:** Department of Neurology, Affiliated Hospital of Shandong University of Traditional Chinese Medicine, Jinan, Shandong, People’s Republic of China

*Dear Editor,*

It was with great interest that we read the article published in the *International Journal of Surgery*. Huang *et al*.^1^ conducted a multicenter retrospective cohort study on patients with an acute, symptomatic, radiologically confirmed acute vertebrobasilar artery occlusion (VBAO) who were treated with endovascular treatment (EVT). Research suggests that unique risk assessment combined with optimization for EVT may enhance long-term prognosis.

VBAO, which causes 1% of all strokes and 5% of major vascular occlusions, is a catastrophic neurological condition with high mortality and severe disability^2^. Its natural history reveals a mortality rate of up to 90% and a significant residual deficit in 65% of patients who do not receive treatment^3^. EVT is now thought to be a viable therapeutic method for acute basilar artery occlusion (ABAO), since standard medical treatment (SMT) alone is linked to low recanalization rates and unfavorable outcomes in patients with ABAO^4^. Since 2006, a number of studies have examined the safety and effectiveness of EVT in individuals with ABAO. The findings of this research indicate that EVT may be linked to improvements in clinical outcomes that are both safe and effective as well as a reduction in patient mortality. Despite being the gold standard treatment for patients with proximal arterial occlusion and anterior circulation acute ischemic stroke, there is currently a dearth of data from randomized clinical trials evaluating the efficacy of EVT for ABAO. It was established by earlier, sizable randomized controlled trials that EVT was not better than conventional medical treatment. Nonetheless, a number of current investigations have demonstrated the effectiveness and safety of EVT in VBAO patients. Therefore, in the actual world, EVT is now the main course of treatment for patients with VBAO.

The purpose of this study was to examine the long-term prognostic variables for patients with VBAO receiving EVT. This innovative method offers wise counsel and motivation for this subject of study’s continued advancement. However, there were certain restrictions. First, a prospective database from 21 centers served as the foundation for the retrospective study design. As a result, while not identical, the criteria used to choose patients, the methodology for the baseline imaging examination, and the choice for either thrombectomy approach varied amongst the centers^5^. Secondly, there’s a chance that some residual confounding factors were missed. Stroke disparities between genders have drawn a lot of attention. Significant gender disparities have been shown in the incidence, clinical presentation, course of treatment, and prognosis of stroke in earlier research^2^.

Large sample size prospective multicenter randomized controlled trials are still required to more extensively evaluate the long-term prognostic variables in VBAO patients treated with EVT in the future. Subgroup analysis and long-term follow-up will be required if more cases are added later.

## Ethical approval

Not applicable.

## Consent

Not applicable.

## Author contribution

J.Z.: study concept or design, data collection, data analysis or interpretation, and writing and revising the paper; H.L. and B.L.: data analysis or interpretation; Z.Z.: data collection; M.H.: revising the paper.

## Conflicts of interest disclosure

The author declares no conflict of interest.

## Research registration unique identifying number (UIN)

This manuscript is a comment. Don’t need UIN.

## Guarantor

Jinping Zhang.

## Data availability statement

This manuscript is a comment. Don’t need a Data availability statement. However, all the data during the current study are publicly available.

## Provenance and peer review

This manuscript is a comment without being invited.
